# Linking genotype to phenotype to identify genetic variation relating to host susceptibility in the mountain pine beetle system

**DOI:** 10.1111/eva.12773

**Published:** 2019-02-19

**Authors:** Catherine I. Cullingham, Rhiannon M. Peery, Colleen E. Fortier, Elizabeth L. Mahon, Janice E. K. Cooke, David W. Coltman

**Affiliations:** ^1^ Department of Biological Sciences University of Alberta Edmonton Alberta Canada; ^2^ Department of Wood Science University of British Columbia Vancouver British Columbia Canada

**Keywords:** adaptive variation, complementary validation, gene expression, *Grosmannia clavigera*, jack pine, lodgepole pine, mountain pine beetle, outlier validation

## Abstract

Identifying genetic variants responsible for phenotypic variation under selective pressure has the potential to enable productive gains in natural resource conservation and management. Despite this potential, identifying adaptive candidate loci is not trivial, and linking genotype to phenotype is a major challenge in contemporary genetics. Many of the population genetic approaches commonly used to identify adaptive candidates will simultaneously detect false positives, particularly in nonmodel species, where experimental evidence is seldom provided for putative roles of the adaptive candidates identified by outlier approaches. In this study, we use outcomes from population genetics, phenotype association, and gene expression analyses as multiple lines of evidence to validate candidate genes. Using lodgepole and jack pine as our nonmodel study species, we analyzed 17 adaptive candidate loci together with 78 putatively neutral loci at 58 locations across Canada (*N* > 800) to determine whether relationships could be established between these candidate loci and phenotype related to mountain pine beetle susceptibility. We identified two candidate loci that were significant across all population genetic tests, and demonstrated significant changes in transcript abundance in trees subjected to wounding or inoculation with the mountain pine beetle fungal associate *Grosmannia clavigera*. Both candidates are involved in central physiological processes that are likely to be invoked in a trees response to stress. One of these two candidate loci showed a significant association with mountain pine beetle attack status in lodgepole pine. The spatial distribution of the attack‐associated allele further coincides with other indicators of susceptibility in lodgepole pine. These analyses, in which population genetics was combined with laboratory and field experimental validation approaches, represent first steps toward linking genetic variation to the phenotype of mountain pine beetle susceptibility in lodgepole and jack pine, and provide a roadmap for more comprehensive analyses.

## 1 INTRODUCTION

Knowledge of adaptive variation is important for many endeavors, such as mitigating the impacts of climate change (Aitken, Yeaman, Holliday, Wang, & Curtis‐McLane, 2008; Jump & Peñuelas, 2005), conserving species (Garcia de Leaniz et al., 2007; Primmer, 2009), and improving value traits in agriculture and forestry (Bruce, Edmeades, & Barker, 2002; Dawson, Lengkeek, Weber, & Jamnadass, 2009; Nelson & Johnsen, 2008). Numerous examples can be cited from the literature in which a population genomics approach is used to detect loci potentially under selection through identifying SNPs with unusual allele frequency patterns across populations and/or environments (Cullingham, Cooke, & Coltman, 2014; Janes et al., 2014; Ojeda Alayon et al., 2017; Tigano, Shultz, Edwards, Robertson, & Friesen, 2017). Despite the popularity of these approaches, challenges remain, including identification of false positives (De Mita et al., 2013; François, Martins, Caye, & Schoville, 2017; Whitlock & Lotterhos, 2015) and linking genotype to phenotype (Stapely et al., 2010; Voelckel, Gruenheit, & Lockhart, 2017).

The false positive discovery rate depends on how well the study system fits the demographic assumptions of the method being used (Excoffier, Hofer, & Foll, 2009; Thornton & Jensen, 2007; Whitlock & Lotterhos, 2015). But, the number of false positives can be reduced by considering loci that have their outlier status verified across multiple statistical approaches (De Mita et al., 2013; Gosset & Bierne, 2012; Narum & Hess, 2011; Nunes, Beaumont, Butlin, & Paulo, 2011). Loci verified in this manner can then be validated by testing for signatures among the same set of loci in different sets of individuals/populations (Bonin, Taberlet, Miaud, & Pompanon, 2006; Stinchcombe & Hoekstra, 2008). Ultimately, however, identification of true positives requires experimental or functional validation (Salvi & Tuberosa, 2005).

Verifying potential candidates by linking genetic variation to phenotype involves manipulating the study system using common gardens, field or laboratory experimentation (Kingsolver, 1996; Vignieri, Larson, & Hoekstra, 2010; Wikelski, Spinney, Schelsky, Scheuerlein, & Gwinner, 2003), quantitative trait locus (QTL) analysis of pedigreed material (Clutton‐Brock & Sheldon, 2010; Slate et al., 2010), or association mapping (Parchman et al., 2012). Many of these options are logistically challenging for nonmodel systems, particularly long‐lived species. An alternative approach is to use multiple lines of investigation, for example, using both gene expression data and population genomics to provide a first indication as to whether genes identified through population genomics have the potential to contribute to adaptive variation (Levy & Borenstein, 2012; Li, Costello, Holloway, & Hahn, 2008; Vasemägi & Primmer, 2005). Positive results obtained with such an analysis can be followed up with more in‐depth functional and sequence analyses of candidate genes. We will term this “complementary validation.” This approach can validate candidates without requiring experimental manipulation of the locus, that is, through targeted mutation or transgenesis of the gene, which is difficult to achieve for many nonmodel species.

Here, we apply a complementary validation approach to study host factors associated with tree defense against *Dendroctonus ponderosae* (Hopkins, mountain pine beetle [MPB]) attack in *Pinus contorta *(Dougl. ex Loud. Var. *latifolia*, lodgepole pine) and *P. banksiana* (Dougl., jack pine). These are sister species that hybridize in north‐central Alberta and the Northwest Territories (Burns et al., submitted; Cullingham, James, Cooke, & Coltman, 2012). Both species are important for forest economy (Law & Valade, 1994; Lee, 1971) and provide habitat for an array of species (Kirk & Hobson, 2001; Martin, Norris, & Drever, 2006), thus making them important study organisms. Since the early 2000s, these tree species have received critical attention due to the ongoing outbreak of MPB. While lodgepole pine is a native host of this opportunistic bark beetle and its fungal associates (Safranyik & Carroll, 2006), the unprecedented scale of this most recent outbreak (Aukema, McKee, Wytrykush, & Carroll, 2016; de la Giroday, Carroll, & Aukema, 2012) has resulted in considerable range expansion of MPB and their microbiome. This range expansion includes spread into naïve lodgepole pine forests (Burke, Bohlmann, & Carroll, 2017; Cudmore, Bjorklund, Carroll, & Lindgren, 2010) and host expansion into the jack pine of Canada's boreal forest (Cullingham et al., 2011). Given the potential for this devastating forest insect pest to establish endemic populations in these novel habitats, understanding how pine susceptibility varies on the landscape is a key component to predicting risk of further spread during this and future outbreaks. Identifying loci that correlate with susceptibility provides one avenue to shed light on this important question.

Despite their ecological and economic importance, lodgepole pine and jack pine are nonmodel species that present considerable challenges for experimental manipulation typically used in more tractable systems to validate candidate genes. Both common gardens and QTL analyses are feasible, but it can take a number of years for trees to reach reproductive maturity that is required for seed collection, and also to reach a sufficient age at which reliable phenotypic measurements can be taken. Given the rapid decay of linkage in *Pinus* genomes, robust QTL analysis requires numerous crosses and dense genetic markers (Neale & Salvolainen, 2004). In addition, genetic transformation of lodgepole and jack pine has never been reported. Therefore, techniques that are often employed to validate candidate genes in model systems like *Arabidopsis thaliana* (Bergelson & Roux, 2010; Brachi et al., 2010) are infeasible for lodgepole and jack pine.

Accordingly, in an effort to identify pine genetic variation that may be of adaptive importance in mediating the host's response to MPB, we have taken advantage of genetic and genomic resources that have been assembled for lodgepole and jack pine (Arango‐Velez et al., 2014; Cullingham, Cooke, & Coltman, 2013; Cullingham et al., 2011; Hall et al., 2013), combining population genomics with gene expression analysis of the host species. We characterized over 800 lodgepole pine, jack pine, and their hybrids across 58 locations at a subset of 96 SNP loci that were previously analyzed at 17 locations (Cullingham et al., 2014). Seventeen of these loci were identified as having statistically significant signatures of local adaptation (Cullingham et al., 2014). Building on these findings, our first objective was to validate these candidates using outlier and environmental correlation analysis similar to Cullingham et al. (2014). Our second objective was to link the validated candidates to MPB attack status of adult trees by comparing genotype frequencies among freshly attacked and unattacked trees sampled within the same stands. Our third objective was to carry out in silico annotation of validated candidate locus sequences to determine whether SNPs were located within protein‐coding or untranslated regions (UTRs), and whether SNPs had the potential to impact protein function. Our final objective was to determine whether candidate loci from the above analyses constituted part of the tree's transcriptomic response to attack by using quantitative reverse transcription–PCR (qRT‐PCR) in response to wounding, and inoculation with the MPB fungal associate *Grosmannia clavigera* ([Robinson‐Jeffrey and Davidson] Zipfel, de Beer and Wingfield; Whitney, 1971). These data and analyses, when combined, provide independent evidence for the putative roles of candidate genes in adaptive traits.

## 2 METHODS

### 2.1 Population samples

**Figure 1:**
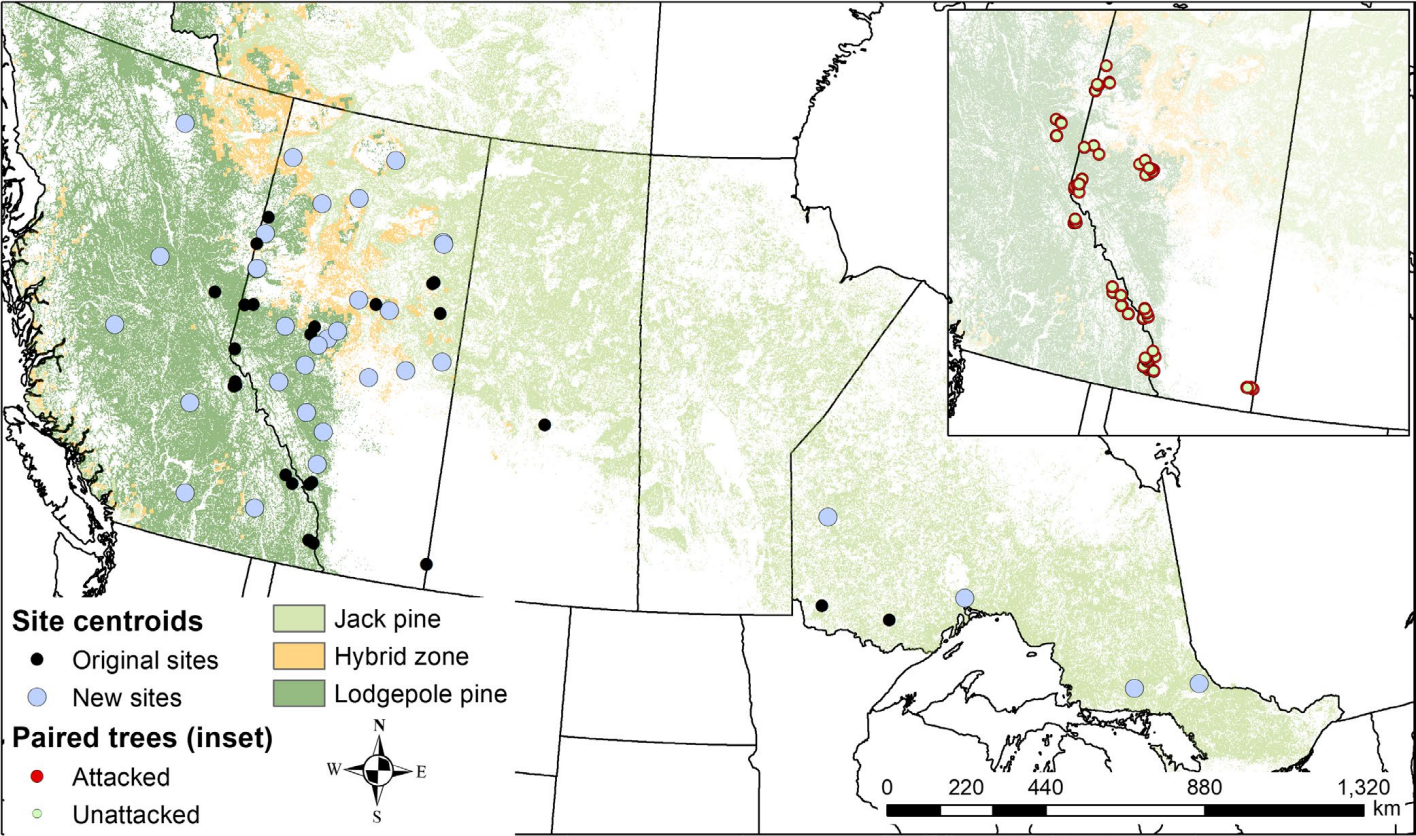
Distribution of sample sites used for this study (“New sites”), together with the sites used in a previous study of lodgepole and jack pine using population genetic methods to identify adaptive candidates (“Original sites,” Cullingham et al., 2014). The inset includes the sampling location of all trees used in a paired analysis to examine whether any of the identified adaptive candidates showed an associated with attack status. Trees with >40 attacks were sampled, and an age‐matched health tree at the same location was also sampled. Predicted distributions of lodgepole, jack pine, and their interspecific hybrids are included for reference (Burns et al., submitted)

Samples used in the population genetics component of this investigation were selected from a larger set of samples used for previous microsatellite (Cullingham et al., 2011, 2012) and SNP (Cullingham et al., 2013) studies, in which foliage was collected from British Columbia, Alberta, Saskatchewan, and Ontario, Canada (*N* = 182 samples from 23 locations). To complement and extend this collection of samples, additional foliage samples were collected in Ontario by the Ontario Ministry of Natural Resources and Forestry (*N* = 137 samples from six locations). Tree locations were determined using GPS. To increase sample coverage in Alberta, we used ex situ conservation seed collections from the Alberta Tree Improvement and Seed Centre, Alberta Agriculture and Forestry (*N* = 507 samples from 38 locations). To obtain seedlings of a sufficient size for analysis, seeds were germinated following the protocol outlined in Cullingham et al. (2012). Germinated seedlings were flash‐frozen in liquid nitrogen and stored at −20°C prior to DNA extraction. GPS locations were collected for field trees, and centroids of locations were used for site‐level analyses (Figure 1).

Twelve of the locations in British Columbia and Alberta were situated within the active MPB outbreak during the 2007–2008 sampling period. At these locations, foliage was sampled from both unattacked and naturally MPB attacked pines. Attacked pines typically had sustained >40 attacks, as determined by counting pitch tubes. In Alberta, attacked trees had been identified the prior autumn via aerial surveys by Alberta Agriculture and Forestry (formerly Alberta Sustainable Resources Development). In all cases, MPB attack status of sampled trees was confirmed by inspecting gallery structure and identification of MPB within galleries. Following identification of a suitable MPB attacked pine for sampling, an unattacked, healthy tree of similar diameter by breast height (DBH) was identified for sampling no more than 250 m from the MPB attacked tree. We refer to these as paired attacked/unattacked trees. Between five and 22 attack/unattacked pairs of trees were collected per location. All field samples were stored on ice or at subzero conditions (in the case of winter sampling) until transported to the laboratory, and were kept at −20°C prior to DNA extraction. Genomic DNA was isolated according to Cullingham et al. (2011).

### 2.2 Genotyping

Cullingham et al. (2013) previously typed 546 lodgepole pine, jack pine, and lodgepole × jack pine hybrids samples from 17 locations at 472 SNP loci using the Golden Gate assay (Illumina, San Diego, CA). From these data, 28 outlying SNP loci were considered adaptive candidates (Cullingham et al., 2014). To validate these findings at a broader scale of locations, we selected 96 of those 472 SNP loci for Sequenom SNP typing (Agena Biosciences, San Diego, CA) including 17 of the adaptive candidates and 79 putatively neutral loci. The adaptive loci we selected as strong candidates were statistically robust (i.e., identified consistently across outlier detection methods) and/or showed changes in transcript abundance in response to *G. clavigera* inoculation in a microarray expression dataset (A. Arango‐Velez, E. L. Mahon, L. M. Galindo‐González, C. E. Fortier, M. J. Meents, C. J.‐T. Ju, W. El Kayal, D. Royko, C. C. J. Copeland, J. E. K. Cooke, unpublished). We included 44 samples from our previous genotyping effort to assess congruence between the two genotyping technologies. The same flanking sequence data used to develop the Illumina SNP chip assay (Supporting Information Table S1) were used to design the Sequenom MassARRAY assay by Agena Biosciences. Genotyping was completed using Sequenom iPLEX Gold Technology at the McGill University and Génome Québec Innovation Centre (Montreal, Canada).

Given that a number of our sample sites occur within the area of hybridization between lodgepole and jack pine, we ran structure (Falush, Stephens, & Pritchard, 2003; Pritchard, Stephens, & Donnelly, 2000) to identify ancestry of all individuals. We used the admixture model, setting the number of clusters (*K*) to two; 500,000 iterations were run, discarding the first 100,000 as burn‐in. We ran the model five times and averaged the *q‐*values across runs to assign ancestry. The cut‐off for pure species was 0.10 ≤ *q* ≥ 0.90; individuals with intermediate values were considered hybrids (Cullingham et al., 2011).

### 2.3 Outlier detection

For consistency, we applied three of the four detection methods that we used in the previous study (Cullingham et al., 2014) together with outflank (Whitlock & Lotterhos, 2015), which was not publicly available at the time of the original publication. We chose not to use matsam (Joost et al., 2007), because it does not incorporate population structure information and results in a high proportion of false positives (Cullingham et al., 2014). For all analyses, we completed outlier detection on the pure species separately. We first used the hierarchical model in arlequin (Excoffier et al., 2009; Excoffier & Lischer, 2010), considering loci to be significant outliers when *p* ≤ 0.01. Second, we used bayescan (Foll & Gaggiotti, 2008), using a burn‐in of 10,000 iterations, thinning interval of 50, sample size of 10,000, and prior odds of 1,000 to limit the number of false positives (Lotterhos & Whitlock, 2014). Third, we used bayenv2 (Coop, Witonsky, Rienzo, & Pritchard, 2010; Gunther & Coop, 2013) to detect loci with significant correlations to the environment. Climate data were obtained from ClimateNA v5.10 (http://tinyurl.com/ClimateNA), which is based on methodology described by Wang, Hamann, Spittlehouse, and Carroll (2016). We used the following variables averaged over a 30‐yr period (1961–1990): temperature (annual temperature, annual maximum temperature, annual minimum temperature, mean temperature wettest quarter, mean temperature driest quarter, mean diurnal range, seasonal temperature, maximum temperature warmest period, minimum temperature coldest period, temperature annual range), precipitation (annual precipitation, precipitation driest period, precipitation wettest period), end of growing season, and start of growing season. We also included longitude, latitude, and elevation. We estimated three covariance matrices to correct for underlying genetic population structure, and ran three iterations with each covariance matrix for every SNP against environmental variables using 50,000 MCMC steps to estimate BayesFactors for each SNP × environment association. We used Jeffrey's scale of evidence (Jeffreys, 1961) to determine the significance of the BayesFactors (3–10 = substantial support, 10–30 = strong support, 30–100 = very strong, >100 = decisive support). Only those loci with consistent values across a majority of iterations were considered. The final method we used was outflank (Whitlock & Lotterhos, 2015). This method estimates the distribution of *F*_ST_ values across loci to better identify statistical outliers by limiting the number of false positives. We verified significant association of loci across the detection methods. Loci that were significant across the majority of methods (≥3 out of the 4 methods), and were previously identified as significant (Cullingham et al., 2014) were considered to be validated candidate loci, and were the focus of further analyses.

### 2.4 Phenotype association

For candidate loci validated using the multiple outlier analysis approaches described above, we compared genotype frequencies between attacked and unattacked trees using a chi‐squared test to identify whether any of the genotypes were associated with attack status. For these analyses, we pooled samples typed in this study (*N* = 47) with previously genotyped trees (*N* = 309, Cullingham et al., 2014), totaling 178 pairs of attacked/unattacked trees across 12 different locations (Figure 1 inset).

### 2.5 In silico sequence analysis

tBLASTx queries of sequence databases (NCBI nr, Altschul, Gish, Miller, Myers, & Lipan, 1990) provided cursory sequence annotation for all of the loci we genotyped (Cullingham et al., 2014). To improve the veracity of the annotation, and to identify whether SNPs in these loci result in a predicted amino acid change, we obtained the coding sequence of the protein of interest from available plant species. These coding sequences were obtained from NCBI GenBank using blastx (Altschul et al., 1990). We used these sequences to generate a more complete alignment and to perform phylogenetic analysis. Alignment was completed using ClustalW (Thompson, Higgins, & Gibson, 1994). The phylogenetic analysis was completed on the aligned deduced amino acid sequences in MEGA6 (Tamura, Stecher, Peerson, Filipski, & Kumar, 2013) using maximum likelihood (ML) with the default settings (bootstrap test of phylogeny, Jones–Taylor–Thorton substitution model, and tree inference using nearest neighbor interchange). Using the alignment, we identified whether the SNP in the validated candidate locus resulted in a predicted amino acid change. If the SNP resulted in a predicted amino acid change, we then generated an in silico predicted three‐dimensional structure of the protein using Phyre^2^ (Kelley, Mezulis, Yates, Wass, & Sternberg, 2015) and SuSPect (Yates, Filippis, Kelley, & Sternberg, 2014) to predict potential impacts of the amino acid change on protein function.

### 2.6 Gene expression analysis samples

To assess whether validated candidates identified may be involved in the physiological response to MPB attack, we analyzed changes in transcript abundance of the candidates in lodgepole and jack pine seedlings inoculated with *G. clavigera*. Seedlings were used for these analyses rather than mature trees to better control for the between‐individual variance in transcript abundance that is typical of experiments that are conducted with nonclonal material. Bark for analysis was harvested from second‐year seedlings of lodgepole pine (Alberta provenance) or jack pine (Ontario provenance) grown under controlled environmental conditions (19°C constant temperature, 20%–25% relative humidity, 16 hr photoperiod with 200 μmol photosynthetically active radiation) and subjected to (a) inoculation with *G. clavigera* spore (M001‐03‐03‐07‐UC04DL09, Roe, Rice, Coltman, Cooke, & Sperling, 2010) suspension applied by pipettor into small holes made in the bark by syringe needle, (b) wounding by syringe needle, or (c) control. Seedlings (*n* = 5–11 replicates per treatment combination) were harvested at 1, 7, and 14 days postinoculation (dpi). Samples were derived from the same randomized complete block design experiment described in detail in Arango‐Velez et al. (2015).

### 2.7 Transcript abundance profiling

Total RNA was extracted from ∼100 mg tissue using the cetyltrimethylammonium bromide (CTAB) protocol of Chang, Puryear, and Cairney (1993) with modifications by Pavy et al. (2008). RNA was quantified with a NanoQuant 200 (Tecan Infinite, Morrisville NC, USA). First strand cDNA was synthesized using Superscript II reverse transcriptase (Invitrogen; Life Technologies, Burlington, ON, Canada), using 2 µg of total RNA treated with DNaseI (Invitrogen; Life Technologies).

To ensure the legitimacy of target and reference genes used for qRT‐PCR, a cDNA corresponding to each sequence was cloned using standard techniques (McAllister et al., 2018), using manually designed primers to promote target specificity (Supporting Information Table S3). Cloned cDNAs were sequenced on a 3,730 DNA analyzer (Thermo Fisher Scientific, Waltham, MA, USA), using the BigDye system (Thermo Fisher Scientific) with T7 (5′‐TAATACGACTCACTATAGGG‐3′) and SP6 (5′‐TATTTAGGTGACACTATAG‐3′) primers. The cDNA sequences are deposited in GenBank under accession numbers MK390469–MK390472.

Sequenced cDNA clones were used for target‐specific qRT‐PCR primer design (Supporting Information Table S3). Quantitative RT‐PCR mixtures (10 μl) consisted of master mix (0.2 mM dNTPs, 0.3 U Platinum Taq Polymerase (Invitrogen), 0.25 Å~ SYBR Green, and 0.1 Å~ ROX), 20 ng cDNA, and 0.8 μM primers. Two technical replicates per biological replicate were analyzed on an ABI PRISM 7900HT Sequence Detection System (Applied Biosystems, Thermo Fisher Scientific, Foster City, CA, USA). The cycling protocol was as follows: 95°C for 2 min followed by 40 cycles of 95°C for 15 s, and 60°C for 1 min. Melting curves were generated using 95°C for 15 s, 60°C for 15 s, and 95°C for 15 s. Amplification efficiencies were estimated using standard curves prepared from serial dilutions of PCR amplicons for each locus and each species. Reference genes were selected from a panel of five genes previously tested on a subset of individuals (32 each of lodgepole and jack pine) from the same growth chamber experiment. Eukaryotic translation initiation factor 5A‐1 (TIF5α) and elongation factor 1 (EFIα) were found to be the most stable across treatments based on analysis of *C_t_* values in BestKeeper v1.0 (Pfaffl, Tichopad, Prgomet, & Neuvians, 2004). Both genes had standard deviation values <1 (lodgepole pine, *SD*_Tif5α_ = 0.45, *SD*_EF1α_ = 0.76; jack pine, *SD*_Tif5α_ = 0.34, *SD*_EF1α_ = 0.47), the correlation between the genes *C_t_* values was significant (*r* = 0.96, *p* < 0.001), and their correlation with the BestKeeper statistic was also significant (*r* > 0.95, *p* = 0.001; Supporting Information Methods & Results), indicating these are stably expressed across treatments. As multiple 384 well plates were required for analyses of all samples, 24 samples (two technical replicates each) were included on all plates as calibrators. Transcript abundance was calculated from *C_t_* values using standard curves.

Statistical analysis of expression data was completed in R v3.4.3 (R Development Core Team, 2017), except where noted. First, we analyzed the *C_t_* data for stability of the reference genes, and to detect outliers using BestKeeper v1.0. Following verification of the reference genes, we used the modeling approach developed by Matz, Wright, and Scott (2013) to analyze the expression data. This method builds on the generalized linear mixed model approach introduced by Steibel, Poletto, Coussens, and Rosa (2009) allowing the incorporation of both fixed and random effects. We used the normalization model in the MCMC.qpcr package (Matz et al., 2013), which is the most powerful model using the information from the reference genes. We included time × treatment as a fixed effect, and sample, genotype (see below), block, and run as random effects. We used a burn‐in period of 10,000 iterations, and an additional 30,000 iterations for data collection, the number of iterations was determined to ensure the model reached consistency.

### 2.8 Allelic resequencing

All lodgepole individuals from the transcript abundance experiment were genotyped at the validated candidates (Lodgc1087 and Lodgc2304) by Sanger sequencing of PCR amplicons. PCR using the same primers as used for qRT‐PCR (Supporting Information Table S3) was carried out in a 25 µl volume with final concentrations of components as follows: 1× buffer, 1 mM dNTP mixture, 0.2 mM each primer, and 1 U of Taq polymerase (New England Biolabs, Whitby, ON). Amplification was performed using a step‐down procedure with an initial denaturation cycle of 94°C for 5 min, followed by loops of two cycles at each annealing temperature starting at 60°C for 2 min, and stepping down the annealing temperature by two degrees per loop to a final temperature of 48°C for the remaining 22 cycles. Amplicons were cleaned using the ExoSAP method (Thermo Fisher Scientific) and sequenced as described above. Resulting sequences were trimmed and SNPs scored using sequencher v. 5.4 (Gene Codes Corporation, Ann Arbor, MI, USA).

## 3 RESULTS

### 3.1 Genotyping

Of the 96 loci included in the Sequenom genotyping assay, 87 were retained following quality control analysis of the genotypes, which included 16 of the 17 adaptive candidate loci. There was a high level of concordance between genotypes returned from the Illumina and Sequenom platforms (98.9% concordant, with all differences being heterozygote vs. homozygote calls). After removing individuals missing>10% of loci, we analyzed genotypes from 849 samples. Using the *q*‐values from structure, 397 were assigned to lodgepole pine, 252 were jack pine, and 200 individuals were assigned to the lodgepole × jack pine hybrid category (hereafter referred to as “hybrid”). For outlier detection, the data were separated into pure lodgepole pine (59 polymorphic loci) and pure jack pine (54 polymorphic loci).

### 3.2 Outlier detection

In lodgepole pine, we identified six significant outlier loci. Of these, only two were verified across three or more of the detection methods and were also detected previously (Cullingham et al., 2014), Lodgc1087 and Lodgc2304 (Table 1). We consider these two loci to be validated candidates. In jack pine, we detected five outlier loci, but these were detected with only one method (arlequin). The validated candidate loci detected in lodgepole pine were monomorphic in jack pine.

**Table 1:** SNP loci identified as a statistical outliers in jack pine and lodgepole pine

Locus	Typed	Outlier detection		Previously identified*	Annotation*
		Lodge	Jack		
Jp_c21224p439	JP + LP			Outlier	Transketolase/dehydrogenase‐5‐phosphate synthase
Jp_c25075p377	JP + LP		A	Outlier	Phenylcoumaran benzylic ether reductase/NmrA‐like negative transcriptional regulator
JpLpc41319p340	M	NA	NA	Outlier	Uncharacterized BCR, YbaB family COG0718
JpLpc47089p1831	JP + LP			Outlier	Dof‐type zinc finger DNA‐binding family protein
JpLpc66545p1207	M	NA	NA	Outlier	Transcribed locus
Lodgc5453p480	JP + LP			Outlier	Nodulin‐like/major facilitator superfamily protein
Lp_c00150p459	JP			Outlier	Circadian clock associated 1
Jp_c24821p611	JP + LP			Outlier	Nodulin MtN3 family protein
Jp_c44637p536	JP			Outlier	Coatomer alpha subunit/glycine‐rich protein
Jp_c44933p494	JP			Outlier	Photosystem II reaction center protein
JpLpc15608p1838	JP			Outlier	Transducin family protein/WD‐40 repeat family protein
JpLpc21364p981	JP		A	Outlier	myb‐like DNA‐binding domain
JpLpc44782p470	JP			Outlier	KNOX1/2 domain/KNOTTED‐like
Lodgc1087p211	LP	A + BE + BS + OF		Outlier	Proteasome alpha type 7
Lodgc2304p514	LP	A + BE + OF		Outlier	Triosephosphate isomerase
Lp_c04318p2154	LP			Outlier	Carbohydrate binding molecule/starch branching enzyme 2.2
Jackc1504p209	JP			Outlier	Transcribed locus
JpLpc30808p665	JP		A		BTB/POZ domain and MATH domain 2
Lodgc15p411	JP + LP		A		YGGT family protein
Lp_c22542p803	JP + LP		A		Glutamate synthase (ferredoxin)/NADH‐dependent
JpLpc45900p1071	LP	A + B			AP2 domain/integrase‐type DNA‐binding superfamily/eukaryotic translation initiation factor
Jp_c31636p544	JP + LP	A			Methylenetetrahydrofolate reductase
Lodgc4306p424	JP + LP	B			Nucleotide‐diphospho‐sugar transferases superfamily
Lodgc4455p208	JP + LP	B			Plant protein of unknown function (DUF868)

Note

Included is information regarding the species they were typed in (JP = jack pine, LP = lodgepole pine, *M* = monomorphic in each species), what tests the loci were significant for, whether the locus was previously detected as an outlier, and their annotation. Loci highlighted in gray were chosen for qRT‐PCR analysis. Abbreviations for outlier detection methods, A, Arelquin, BS, BayeScan, BE, Bayenv, O, Outflank.

* Cullingham et al. (2014)

### 3.3 Phenotype association

**Figure 2:**
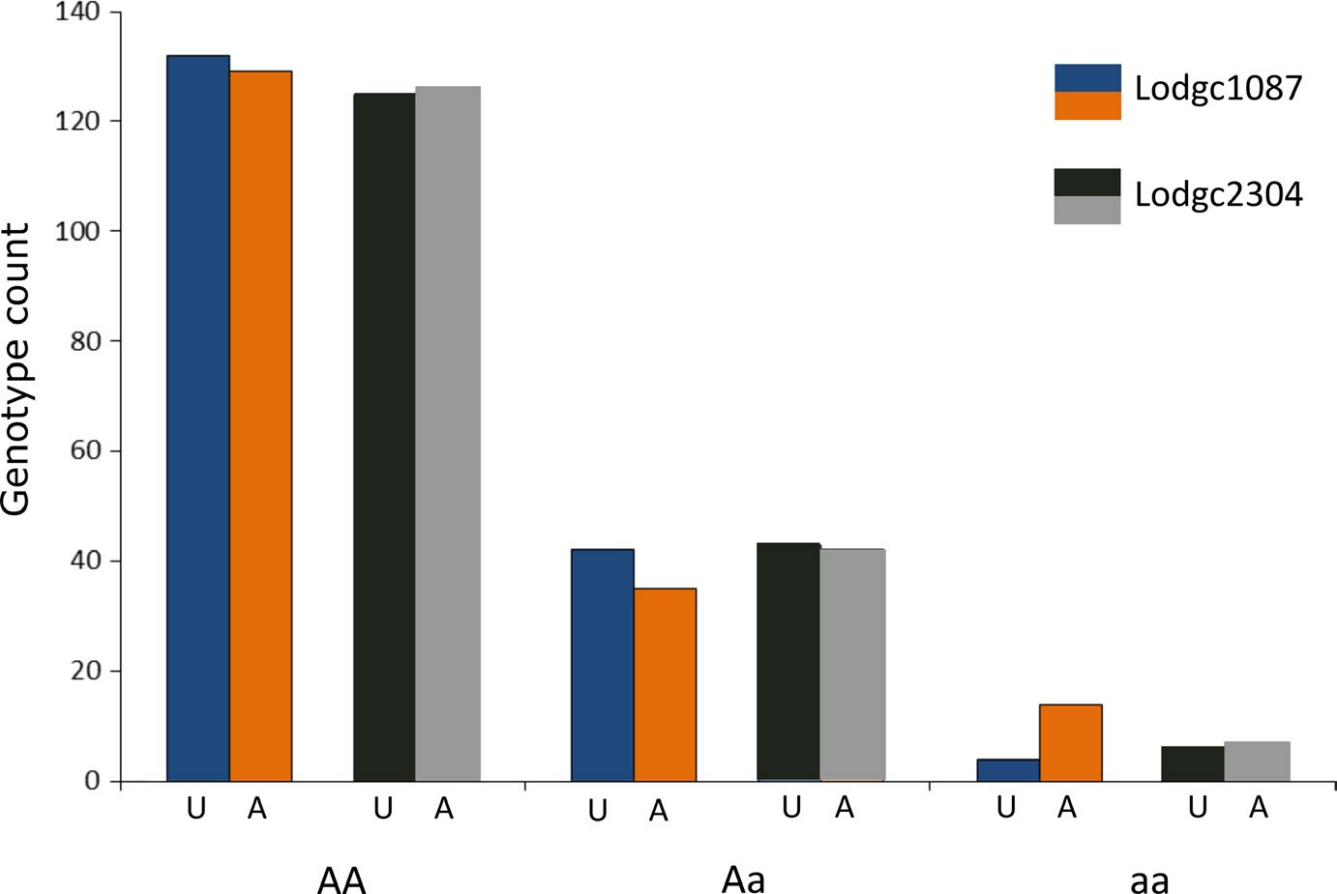
Distribution of genotypes (A—major allele, a—minor allele) that were observed for lodgepole pine individuals recently attacked by mountain pine beetle (“A”), paired with equal aged, location matched unattacked (“U”) lodgepole pine at two validated candidate loci, Lodgc1087 and Lodgc2304 (*N* = 178 pairs). Trees were obtained from 12 different locations in Alberta and British Columbia (Figures 1 and 3)

**Figure 3:**
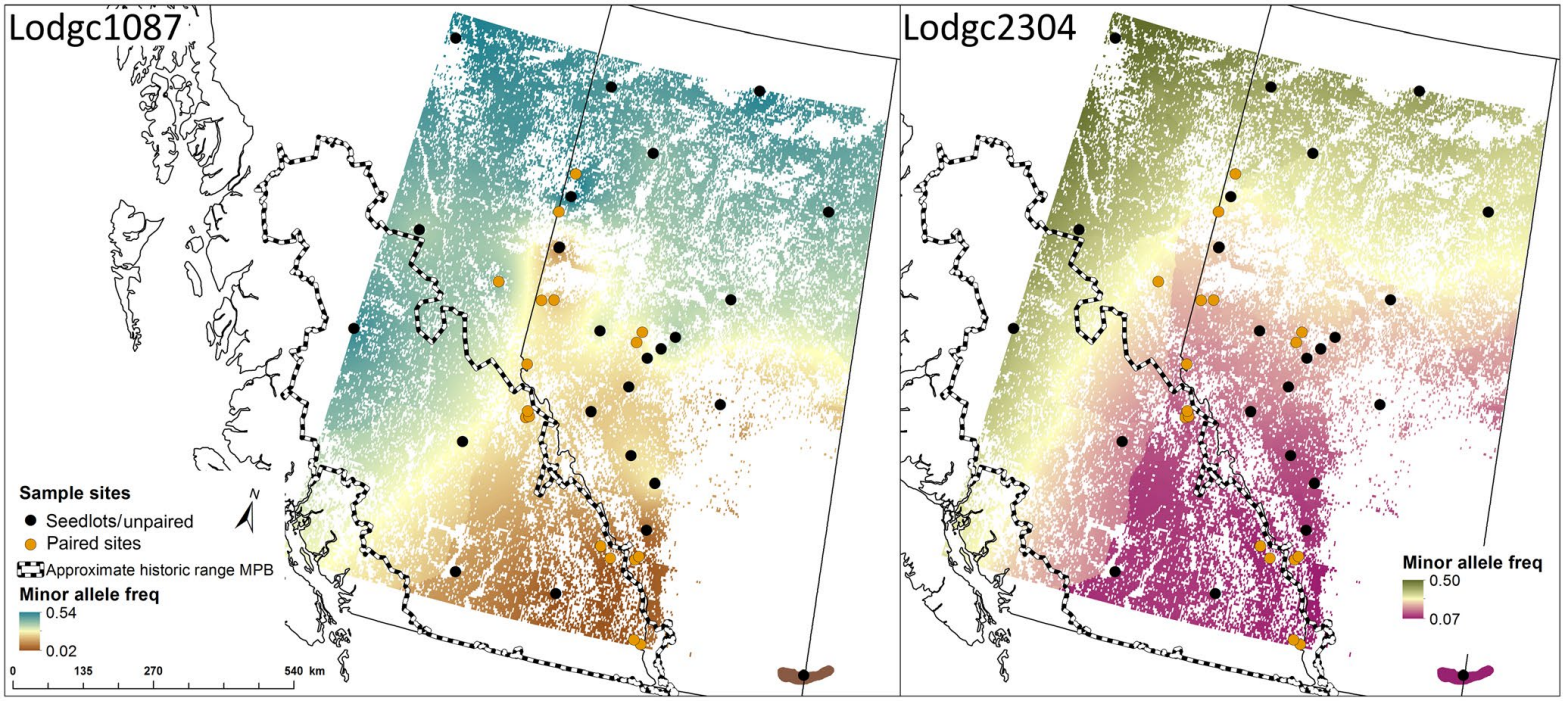
Interpolated map of the minor allele for Lodgc1087 and Lodgc2304 across sample sites corrected for pine distribution (Yemshanov, McKenney, and Pedlar (2012). Sites that were used to assess relationship of loci with attack status are indicated as “paired sites.” Included in the map is an approximation of the historic range of mountain pine beetle based on attack data from 1959 to 1999 (British Columbia Ministry of Forests, Lands, and Natural Resources [http//www.for.gov.bc.ca/ftp/HFP/external/!publish/])

We found an association with successful MPB attack at one of the two validated candidate loci. For Lodgc1087, genotype was significantly associated with attack status ($\chi_{df\;=\;2}^{2}$ = 6.23, *p* = 0.044), where the homozygous recessive genotype was more likely to be attacked than expected (Figure 2). We found no significant association for Lodgc2304 ($\chi_{df\;=\;2}^{2}$ = 0.089, *p* = 0.956). Association in jack pine was not tested as these two loci were monomorphic in jack pine. To understand the spatial distribution of allelic variation at the two validated candidates, we created interpolated maps of the frequency of the minor allele using all genotype data in Alberta and British Columbia (this study and Cullingham et al., 2014). Interpolation was completed using the kriging option in ArcMap 10.5 (Figure 3).

### 3.4 In silico sequence analysis

The derived amino acid sequence for contig Lodgc1087 aligned with high similarity (~20% divergence) to proteasome subunit α_7_ from a range of angiosperm species (e.g., *Prunus mume*, *Vitis vinifera*, *Fragaria vesca*, and *Glycine max*; Supporting Information Figure S1). The candidate SNP was within the coding region and conferred an amino acid change from glutamic acid to aspartic acid, both belong to the acidic amino acid group. This amino acid change is relatively common across species and does not likely result in a change in protein function based on the prediction analysis in Phyre^2^ and SuSPect. Contig Lodgc2304 showed highest sequence similarity to the plastid triosephosphate isomerase (pTPI), which is encoded in the nuclear genome. We confirmed this was the plastid and not cytosolic version of this protein by aligning with both isoforms from a number of species (Supporting Information Figure S2). Based on the amino acid alignment, the outlier SNP was within the coding region and causes an amino acid change from glycine to alanine. These are functionally different amino acids, and the prediction/mutation analysis in Phyre^2^ and SuSPect suggested a moderate probability of a change in protein function.

### 3.5 Transcript abundance profiling

**Figure 4:**
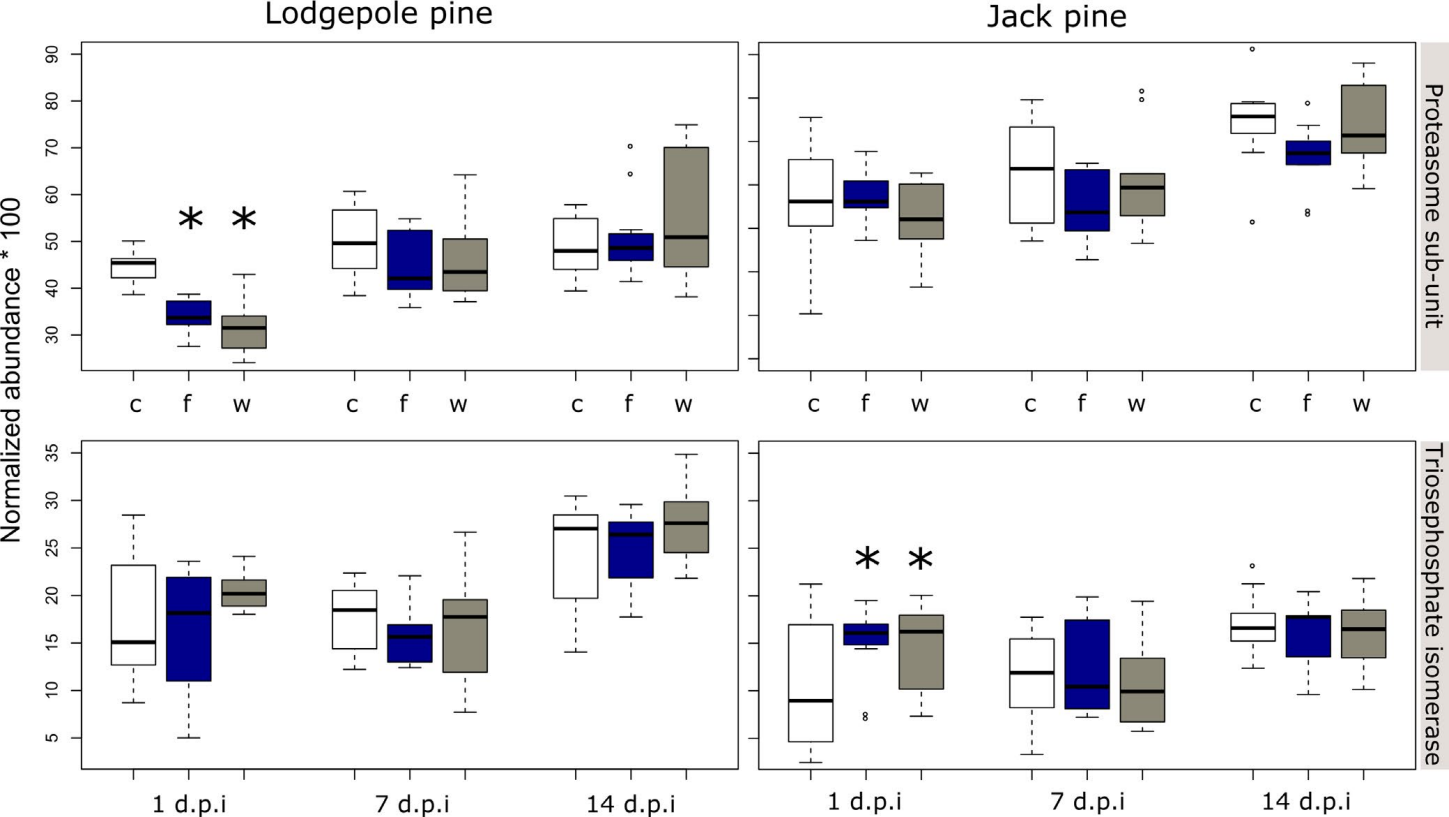
Normalized transcript abundance levels for proteasome subunit α7 (“Proteasome subunit”) and plastid triosephosphate isomerase (“Triosephosphate isomerase”) under three conditions (c, control; f, fungal inoculation with *Grosmannia clavigera*; and w, wound), across time (1, 7, and 14 dpi). Significant fold changes are indicated by an “*,” where *p* ≤ 0.05. Normalized abundance was estimated by dividing molecule abundance for the target gene by the geometric mean of the reference genes Tif5α, and EF1α

Quantitative RT‐PCR with transcript‐specific primers was used to measure transcript abundance corresponding to proteasome sub‐unit α7 and pTPI in lodgepole and jack pine seedlings challenged with *G. clavigera*. We identified four outlier samples in lodgepole pine and three in jack pine using the sample‐based fold‐change analysis in BestKeeper. Following outlier removal, we statistically compared expression levels across treatments for each species using the normalization Bayesian model in the MCMC.qpcr R package. For lodgepole pine, proteasome subunit α7 had significantly lower transcript abundance for both wounding (*p* = 0.002) and fungal treatment (*p* = 0.006) on day 1 postinoculation (Figure 4). For jack pine, we found pTPI had significantly higher transcript abundance for both wounding (*p* = 0.010) and fungal treatment (*p* = 0.012) on day 1 postinoculation (Figure 4). No other treatment × time combinations were significant.

## 4 DISCUSSION

While studies using population genomics to identify candidate loci are on the rise, we are not yet seeing a concomitant increase in validation studies (Shafer et al., 2015). Many studies use functional annotation to validate candidates, which has been shown to be highly biased (Pavlidis, Jensen, Stephan, & Stamatakis, 2012). Examining previously identified candidate loci in pine (Cullingham et al., 2014), across a larger set of samples representing a greater geographic distribution, we have validated two loci within protein‐coding genes that may relate to host response to MPB. We have used multiple lines of evidence including independent natural, and experimental populations as part of this validation. We have called this approach complementary validation. Through this approach, we have identified a locus in lodgepole pine that could be used, in conjunction with other markers, to predict host susceptibility to MPB, and defined an analytical pipeline to identify additional genetic loci that contribute to host susceptibility.

Finding links between genetic variation and phenotype is a challenging problem in evolutionary biology (Naish & Hard, 2008). We have taken a unique approach to addressing this question, considering attack status within a natural outbreak to represent a phenotype of MPB susceptibility. Through the analysis of paired attacked and unattacked lodgepole pine trees, we have found a significant association with genotype at one of the validated candidates, Lodgc1087 with MPB attack status. To our knowledge, this is the first report of a pine locus associated with MPB host susceptibility in lodgepole pine. Interestingly, jack pine populations do not carry the risk‐associated polymorphism for Lodgc1087.

The landscape‐level spatial distribution of the attack‐associated allele for Lodgc1087 in lodgepole pine corresponds to the historic MPB climatic suitability classifications (Burke et al., 2017). The allele is at a high frequency where MPB climatic suitability has historically been low or very low (Figure 3), while the frequency of the allele is lowest in regions with very high MPB suitability. Forests classified as having extreme historic climatic suitability exhibit the highest likelihood for four determinants of MPB success: completion of a 1‐year life cycle, overwintering temperatures favorable for larval survival, advantageous emergence and dispersal conditions, and spring precipitation as a proxy for tree defense capacity (Burke et al., 2017; Safranyik, Shrimpton, & Whitney, 1975).

Based on population genetics studies of MPB and lodgepole pine (Bentz et al., 2010; Cwynar & MacDonald, 1987; Mock et al., 2007; Wheeler & Guries, 1982) Burke et al. (2017) suggested that the northward post‐glacial recolonization of western North America from southern refugia by MPB and lodgepole pine has been asynchronous, meaning that while southern populations of lodgepole pine are likely to have shared a long co‐evolutionary history with MPB, northern populations of lodgepole pine are likely to have not. Burke et al. (2017) proposed that the spatial gradient of historic climatic suitability can serve as a proxy for the degree of evolutionary association between MPB and lodgepole pine. Under this assumption, we postulate that the southern lodgepole pine populations located in high or extreme historic climatic conditions have experienced the greatest degree of evolutionary selective pressure from MPB, resulting in reduced frequencies of the attack‐associated minor allele. Conversely, northern populations in which this attack‐associated minor allele is present at relatively high frequencies have experienced no selection pressure from MPB.

In silico annotations suggest that both candidates participate in pathways involved in tree responses to environment, and therefore conceivably play direct or indirect roles in tree defense. Lodgc1087, the locus associated with MPB attack status, is within a gene encoding one of the proteasome alpha subunits of the 26S proteasome (Fu et al., 1999). The ubiquitin‐26S proteasome machinery functions in targeted protein breakdown, and is highly conserved across eukaryotes (Dreher & Callis, 2007). The protein‐coding sequence containing Lodgc1087 exhibited the highest similarity to proteasome subunit α_7_, which acts as a gate to the catalytic chamber of the 20S core protease (Book et al., 2010). Because plants are sessile, they have evolved complex proteolytic systems to manage responses to biotic stresses such as herbivore and pathogen attack, and the proteasome‐ubiquitin pathway is considered an important component of these response systems (Dielen, Badaoui, Candresse, & German‐Retana, 2010; Small & Vierstra, 2004; Vierstra, 2009). The 20S core protease has been implicated in plant immunity to pathogens (Üstün et al., 2016) and also plays an important role in defense‐associated hormone signaling (Vierstra, 2009).

Lodgc2304 is within pTPI, which codes for a key plastid enzyme that is encoded in the nuclear genome (Henze, Schnarrenberger, Kellermann, & Martin, 1994). Plastid TPI interconverts dihydroxyacetone phosphate to glyceraldehyde 3‐phosphate, controlling the triose phosphate pool. Plastid TPI and the triose phosphate pool are integral to the Calvin cycle of photosynthesis (Raines, 2003) and to glycolysis (Plaxton, 1996). Glycolysis occurs in both the cytosol and plastids in plants (Plaxton, 1996). Triose phosphates generated by pTPI are central to carbon metabolism in plants, serving as the photosynthesis‐derived carbohydrate skeletons for anabolic synthesis of more complex carbohydrates such as the hexoses, disaccharides, and starch, as well as the glycolytic intermediates for catabolic energy production (Plaxton, 1996; Raines, 2003). These processes are critical for the carbon‐ and energy‐intensive demands of plant defenses. Changes in pTPI gene expression have been found in response to fungal inoculation in cabbage (*Brassica carinata*; Subramanian, Bansal, & Kay, 2005), and beech trees (*Fagus grandifolia* Ehrh; Mason, Koch, Krasowski, & Loo, 2013).

We next examined whether these genes were implicated in lodgepole and jack pine defense responses by comparing transcript abundance under control and induced conditions. Given the logistical challenges associated with conducting gene expression analyses on MPB attacked pines, we used *G. clavigera* inoculation as a proxy to elicit an MPB defense response. We used a novel Bayesian mixed‐effect model (Matz et al., 2013) to analyze the transcript abundance data. These analyses revealed that transcript abundance corresponding to proteasome subunit α_7_ significantly decreased in lodgepole pine—but not in jack pine—shortly after fungal inoculation. These data suggest that this gene, harboring the Lodgc1087 attack‐associated SNP, may play some role in host susceptibility. In contrast, transcript abundance corresponding to pTPI showed a significant increase in response to both wounding and fungal inoculation at early time points in jack pine, but not lodgepole pine. As this experiment was not explicitly designed to test gene expression differences in Lodgc1087 genetic variants, we are unable to establish a link between gene expression and the mutation. Ongoing experiments will address this question in both seedling and mature tree material.

## 5 CONCLUSIONS

In this study, we have used complementary validation appropriate for nonmodel organisms by incorporating evidence from multiple approaches to identify genetic variation potentially contributing to differential responses in lodgepole and jack pine trees to MPB and their fungal associates. While Cullingham et al. (2014) used a conservative approach to identify the initial candidate outlier list, only two of the originally identified candidates were validated, suggesting the detection of false positives. The original set of loci was significant across multiple detection methods, which in theory should have greatly reduced the proportion of false outliers (De Mita et al., 2013; Gosset & Bierne, 2012; Narum & Hess, 2011; Nunes et al., 2011). Given we validated only two candidates that were previously identified using stringent criteria, should serve as a precaution for studies attempting to identify adaptive candidates without proper validation as a consideration (Meirmans, 2015).

Of the two validated candidates, one shows promise as a marker for increased susceptibility to MPB attack. We have identified a clear association between attack status and genotype for the Lodgc1087‐containing gene encoding proteasome subunit α_7_ in lodgepole pine, and provided experimental evidence that this gene is involved in the defense response. This is the first report of a locus associated with pine host susceptibility, of which genetic resistance is one component. Genetic resistance to MPB has been reported to be moderately heritable in lodgepole pine (Yanchuk, Murphy, & Wallin, 2008), but resistance to this herbivore likely comprises hundreds—if not thousands—of loci with potentially small effects. Genotype‐by‐environment effects are also likely to be substantive. Nevertheless, our finding of an association of Lodg1087 with attack status demonstrates the utility of a population genomics approach to defining the genetic component of pine host suitability for MPB, and is a meaningful first step toward delineating a susceptible vs. resilient host genotype. This approach has been adopted as part of a comprehensive, large‐scale effort to identify additional loci associated with attack status that are also implicated in pine defense responses. Our goal is to develop models that will better define the linkages between host genotype and attack probability. Such models can be used for applications such as refining stand susceptibility indices used in decision support systems (Cooke & Carroll, 2017), as well as in tree improvement and ex situ conservation programs to develop more resilient forests.

## CONFLICT OF INTEREST

None declared.

## Data Availability

Genotype data are available through the University of Alberta Dataverse data repository (https://doi.org/10.7939/DVN/O1ZG1P), sequences of cloned genes are deposited on GenBank (MK390469–MK390472), and flanking sequences of SNPs are included in the Supporting Information Table S1).

## References

[eva12773-bib-0001] Aitken, S. N. , Yeaman, S. , Holliday, J. A. , Wang, T. , & Curtis‐McLane, S. (2008). Adaptation, migration, extirpation, climate change outcomes for tree populations. Evolutionary Applications, 1, 95–111. 25567494 10.1111/j.1752-4571.2007.00013.x PMC3352395

[eva12773-bib-0002] Altschul, S. F. , Gish, W. , Miller, W. , Myers, E. W. , & Lipan, D. J. (1990). Basic local alignment search tool. Journal of Molecular Biology, 215, 403–410. 10.1016/S0022-2836(05)80360-2 2231712

[eva12773-bib-0003] Arango‐Velez, A. , El Kayal, W. , Copeland, C. C. J. , Zaharia, L. I. , Lusebrink, I. , & Cooke, J. E. K. (2015). Differences in defense response of *Pinus contorta* and *Pinus banksiana* to the mountain pine beetle fungal associate *Grosmania clavigera* are affected by water deficit. Plant, Cell & Environment, 39, 726–744. 10.1111/pce.12615 26205849

[eva12773-bib-0004] Arango‐Velez, A. , Galindo Gonzalez, L. M. , Meents, M. J. , El Kayal, W. , Cooke, B. J. , Linsky, J. , … Cooke, J. E. K. (2014). Influence of water deficit on the molecular responses of *Pinus contorta* × *Pinus banksiana* mature trees to infection by the mountain pine beetle fungal associate, *Grosmania clavigera* . Tree Physiology, 34, 1220–1239. 24319029 10.1093/treephys/tpt101 PMC4277265

[eva12773-bib-0005] Aukema, B. H. , McKee, F. R. , Wytrykush, D. L. , & Carroll, A. L. (2016). Population dynamics and epidemiology of four species of *Dendroctonus* (Coleoptera, Curculionidae), 100 years since J.M. Swaine. Canadian Entomologist, 148, S82–S110. 10.4039/tce.2016.5

[eva12773-bib-0006] Bentz, B. J. , Régnière, J. , Fettig, C. J. , Hansen, E. M. , Hayes, J. L. , Hicke, J. A. , … Seybold, S. J. (2010). Climate change and bark beetles of the western United States and Canada: Direct and indirect effects. BioScience, 60, 602–613. 10.1525/bio.2010.60.8.6

[eva12773-bib-0007] Bergelson, J. , & Roux, F. (2010). Towards identifying genes underlying ecologically relevant traits in *Arabidopsis thaliana* . Nature Reviews Genetics, 11, 867–879. 10.1038/nrg2896 21085205

[eva12773-bib-0008] Bonin, A. , Taberlet, P. , Miaud, C. , & Pompanon, F. (2006). Explorative genome scan to detect candidate loci for adaptation along a gradient of altitude in the common frog (*Rana temporaria*). Molecular Biology and Evolution, 23, 773–783. 10.1093/molbev/msj087 16396915

[eva12773-bib-0009] Book, A. J. , Gladman, N. P. , Lee, S. S. , Scalf, M. , Smith, L. M. , & Vierstra, R. D. (2010). Affinity purification of the Arabidopsis 26S proteasome reveals a diverse array of plant proteolytic complexes. Journal of Biological Chemistry, 285, 25554–25569. 20516081 10.1074/jbc.M110.136622 PMC2919120

[eva12773-bib-0010] Brachi, B. , Faure, N. , Horton, M. , Flahauw, E. , Vazquez, A. , Nordborg, M. , … Roux, F. (2010). Linkage and association mapping of *Arabidopsis thaliana* flowering time in nature. PLoS Genetics, 6, e10000940 10.1371/journal.pgen.1000940 PMC2865524 20463887

[eva12773-bib-0011] Bruce, W. B. , Edmeades, G. O. , & Barker, T. C. (2002). Molecular and physiological approaches to maize improvement for drought tolerance. Journal of Experimental Biology, 53, 13–25. 11741036

[eva12773-bib-0012] Burke, J. L. , Bohlmann, J. , & Carroll, A. L. (2017). Consequences of distributional asymmetry in a warming environment, invasion of novel forests by the mountain pine beetle. Ecosphere, 8, e01778 10.1002/ecs2.1778

[eva12773-bib-0013] Burns, I. , James, P. M. A. , Coltman, D. W. , & Cullingham, C. I. (submitted). Spatial and genetic structure of the lodgepole x jack pine hybrid zone. Canadian Journal of Forest Research. cjfr‐2018‐0428.

[eva12773-bib-0014] Chang, S. , Puryear, J. , & Cairney, J. (1993). Simple and efficient method for isolating RNA from pine trees. Plant Molecular Biology Reporter, 11, 113–116.

[eva12773-bib-0015] Clutton‐Brock, T. , & Sheldon, B. C. (2010). Individuals and populations: The role of long‐term, individual based studies of animals in ecology and evolutionary biology. Trends in Ecology and Evolution, 25, 562–573. 10.1016/j.tree.2010.08.002 20828863

[eva12773-bib-0016] Cooke, B. J. , & Carroll, A. L. (2017). Predicting the risk of mountain pine beetle spread to eastern pine forests: Considering uncertainty in uncertain times. Forest Ecology and Management, 396, 11–25. 10.1016/j.foreco.2017.04.008

[eva12773-bib-0017] Coop, G. , Witonsky, D. , Di Rienzo, A. , & Pritchard, J. K. (2010). Using environmental correlations to identify loci underlying local adaptation. Genetics, 185, 1411–1423. 10.1534/genetics.110.114819 20516501 PMC2927766

[eva12773-bib-0018] Cudmore, T. J. , Bjorklund, N. , Carroll, A. L. , & Lindgren, B. S. (2010). Climate change and range expansion of an aggressive bark beetle: Evidence of higher reproduction in naïve host tree populations. Journal of Applied Ecology, 47, 1036–1043. 10.1111/j.1365-2664.2010.01848.x

[eva12773-bib-0019] Cullingham, C. I. , Cooke, J. E. K. , & Coltman, D. W. (2013). Effects of introgression on the genetic population structure of two ecologically and economically important conifer species, lodgepole pine (*Pinus contorta* var. *latifolia*) and jack pine (*Pinus banksiana*). Genome, 56, 577–585. 24237338 10.1139/gen-2013-0071

[eva12773-bib-0020] Cullingham, C. I. , Cooke, J. E. K. , & Coltman, D. W. (2014). Cross‐species outlier detection reveals different evolutionary pressures between sister species. New Phytologist, 204, 215–229. 10.1111/nph.12896 24942459 PMC4260136

[eva12773-bib-0021] Cullingham, C. I. , Cooke, J. E. K. , Dang, S. , Davis, C. S. , Cooke, B. J. , & Coltman, D. W. (2011). Mountain pine beetle host‐range expansion threatens the boreal forest. Molecular Ecology, 20, 2157–2171. 10.1111/j.1365-294X.2011.05086.x 21457381 PMC3116149

[eva12773-bib-0022] Cullingham, C. I. , James, P. M. A. , Cooke, J. E. K. , & Coltman, D. W. (2012). Characterizing the physical and genetic structure of the lodgepole pine × jack pine hybrid zone, mosaic structure and differential introgression. Evolutionary Applications, 5, 879–891. 10.1111/j.1752-4571.2012.00266.x 23346232 PMC3552405

[eva12773-bib-0023] Cwynar, L. C. , & MacDonald, G. M. (1987). Geographical variation of lodgepole pine in relation to population history. American Naturalist, 129, 463–469. 10.1086/284651

[eva12773-bib-0024] Dawson, I. K. , Lengkeek, A. , Weber, J. C. , & Jamnadass, R. (2009). Managing genetic variation in tropical trees, linking knowledge with action in agroforestry ecosystems for improved conservation and enhanced livelihoods. Biodiversity and Conservation, 18, 969–986. 10.1007/s10531-008-9516-z

[eva12773-bib-0025] de la Giroday, H. M. C. , Carroll, A. L. , & Aukema, B. H. (2012). Breach of the northern Rocky Mountain geoclimatic barrier, initiation of range expansion by the mountain pine beetle. Journal of Biogeography, 39, 1112–1123. 10.1111/j.1365-2699.2011.02673.x

[eva12773-bib-0026] De Mita, S. , Thuillet, A.‐C. , Gay, L. , Ahmadi, N. , Manel, S. , Ronfort, J. , & Vigouroux, Y. (2013). Detecting selection along environmental gradients, analysis of eight methods and their effectiveness for outbreeding and selfing populations. Molecular Ecology, 22, 1383–1399. 10.1111/mec.12182 23294205

[eva12773-bib-0027] Dielen, A.‐S. , Badaoui, S. , Candresse, T. , & German‐Retana, S. (2010). The ubiquitin/26S proteasome system in plant‐pathogen interactions, a never‐ending hide and seek game. Molecular Plant Pathology, 11, 293–308. 10.1111/j.1364-3703.2009.00596.x 20447278 PMC6640532

[eva12773-bib-0028] Dreher, K. , & Callis, J. (2007). Ubiquitin, hormones and biotic stress in plants. Annals of Botany, 99, 787–822. 10.1093/aob/mcl255 17220175 PMC2802907

[eva12773-bib-0029] Excoffier, L. , Hofer, T. , & Foll, M. (2009). Detecting loci under selection in a hierarchically structured population. Heredity, 103, 285–298. 10.1038/hdy.2009.74 19623208

[eva12773-bib-0030] Excoffier, L. , & Lischer, H. E. L. (2010). Arelquin suite ver 3.5, A new series of programs to perform population genetic analyses under Linux and Windows. Molecular Ecology Resources, 10, 564–567. 21565059 10.1111/j.1755-0998.2010.02847.x

[eva12773-bib-0031] Falush, D. , Stephens, M. , & Pritchard, J. K. (2003). Inference of population structure using multilocus genotype data, linked loci and correlated allele frequencies. Genetics, 164, 1567–1587. 12930761 10.1093/genetics/164.4.1567 PMC1462648

[eva12773-bib-0032] Foll, M. , & Gaggiotti, O. (2008). A genome‐scan method to identify selected loci appropriate for both dominant and codominant markers, a Bayesian perspective. Genetics, 180(2), 977–993. 10.1534/genetics.108.092221 18780740 PMC2567396

[eva12773-bib-0033] François, O. , Martins, H. , Caye, K. , & Schoville, S. (2017). Controlling false discoveries in genome scans for selection. Molecular Ecology, 25, 454–469. 10.1111/mec.13513 26671840

[eva12773-bib-0034] Fu, H. , Girod, P.‐A. , van Doelling, J. H. , Nocker, S. , Hochstrasser, M. , Finley, D. , & Vierstra, R. D. (1999). Structure and function analysis of 26S proteasome subunits from plants. Molecular Biology Reports, 26, 137–146. 10363660 10.1023/a:1006926322501

[eva12773-bib-0035] Garcia de Leaniz, C. , Fleming, I. A. , Einum, S. , Verspoor, E. , Jordan, W. C. , Consugra, S. , … Quinn, T. P. (2007). A critical review of adaptive genetic variation in Atlantic salmon, implications for conservation. Biological Reviews, 82, 173–211. 10.1111/j.1469-185X.2006.00004.x 17437557

[eva12773-bib-0036] Gosset, C. C. , & Bierne, N. (2012). Differential introgression from a sister species explains high FST outlier loci within a mussel species. Journal of Evolutionary Biology, 26, 14–26. 10.1111/jeb.12046 23199184

[eva12773-bib-0037] Gunther, T. , & Coop, G. (2013). Robust identification of local adaptation from allele frequencies. Genetics, 195, 205–220. 10.1534/genetics.113.152462 23821598 PMC3761302

[eva12773-bib-0038] Hall, D. E. , Yuen, M. M. S. , Jancsik, S. , Lara Quesada, A. , Dullat, H. K. , Li, M. , … Bohlmann, J. (2013). Transcriptome resources and functional characterization of monoterpene synthases for two host species of the mountain pine beetle, lodgepole pine (*Pinus contorta*) and jack pine (*Pinus banksiana*). BMC Plant Biology, 13, 80 10.1186/1471-2229-13-80 23679205 PMC3668260

[eva12773-bib-0039] Henze, K. , Schnarrenberger, C. , Kellermann, J. , & Martin, W. (1994). Chloroplast and cytosolic triosephosphate isomerases from spinach, purification, microsequencing and cDNA cloning of the chloroplast enzyme. Plant Molecular Biology, 26, 1961–1973. 10.1007/BF00019506 7858230

[eva12773-bib-0040] Janes, J. K. , Li, Y. , Keeling, C. I. , Yuen, M. M. S. , Boone, C. K. , Cooke, J. E. K. , Bohlmann, J. , … Sperling, F. A. H. (2014). How the mountain pine beetle (*Dendroctonus ponderosae*) breached the Canadian Rocky mountains. Molecular Biology and Evolution, 31(7), 1803–1815. 10.1093/molbev/msu135 24803641 PMC4069619

[eva12773-bib-0041] Jeffreys, H. (1961). Theory of probability (p. 432). Oxford, UK: Oxford University Press.

[eva12773-bib-0042] Joost, S. , Bonin, A. , Bruford, M. W. , Després, L. , Conord, C. , Erhardt, G. , & Taberlet, P. (2007). A spatial analysis method (SAM) to detect candidate loci for selection, towards a landscape genomic approach to adaptation. Molecular Ecology, 16, 3955–3969. 17850556 10.1111/j.1365-294X.2007.03442.x

[eva12773-bib-0043] Jump, A. S. , & Peñuelas, J. (2005). Running to stand still, adaptation and the response of plants to rapid climate change. Ecology Letters, 8, 1010–1020. 10.1111/j.1461-0248.2005.00796.x 34517682

[eva12773-bib-0044] Kelley, L. A. , Mezulis, S. , Yates, C. M. , Wass, M. N. , & Sternberg, M. J. E. (2015). The Phyre2 web portal for protein modelling, prediction and analysis. Nature Protocols, 10, 845–858. 25950237 10.1038/nprot.2015.053 PMC5298202

[eva12773-bib-0045] Kingsolver, J. G. (1996). Experimental manipulation of wing pigment pattern and survival in western white butterflies. American Naturalist, 147, 296–306. 10.1086/285852

[eva12773-bib-0046] Kirk, D. A. , & Hobson, K. A. (2001). Bird‐habitat relationships in jack pine boreal forests. Forest Ecology and Management, 147, 217–243. 10.1016/S0378-1127(00)00465-5

[eva12773-bib-0047] Law, K.‐N. , & Valade, J. L. (1994). Status of the utilization of jack pine (*Pinus banksiana*) in the pulp and paper industry. Canadian Journal of Forest Research, 24, 2078–2084.

[eva12773-bib-0048] Lee, Y. (1971). Predicting mortality for even‐aged stands of lodgepole pine. Forestry Chronicle, 47, 29–32. 10.5558/tfc47029-1

[eva12773-bib-0049] Levy, R. , & Borenstein, E. (2012). Reverse ecology, from systems to environments and back In Soyer O. S. (Ed.), Evolutionary systems biology (pp. 329–345). New York, NY: Springer. 10.1007/978-1-4614-3567-9_15 22821465

[eva12773-bib-0050] Li, Y. F. , Costello, J. C. , Holloway, A. K. , & Hahn, M. W. (2008). “Reverse ecology” and the power of population genomics. Evolution, 62, 2984–2994. 18752601 10.1111/j.1558-5646.2008.00486.x PMC2626434

[eva12773-bib-0051] Lotterhos, K. E. , & Whitlock, M. C. (2014). Evaluation of demographic history and neutral parameterization on the performance of F_ST_ outlier tests. Molecular Ecology, 23, 2178–2192. 24655127 10.1111/mec.12725 PMC4228763

[eva12773-bib-0052] Martin, K. , Norris, A. , & Drever, M. (2006). Effects of bark beetle outbreaks on avian biodiversity in the British Columbia interior, implications for critical habitat management. Journal of Ecosystems and Management, 7, 10–24.

[eva12773-bib-0053] Mason, M. E. , Koch, J. L. , Krasowski, M. , & Loo, J. (2013). Comparisons of protein profiles of beech bark disease resistant and susceptible American beech (*Fagus grandifolia*). Proteome Science, 11, 2 10.1186/1477-5956-11-2 23317283 PMC3575302

[eva12773-bib-0054] Matz, M. V. , Wright, R. M. , & Scott, J. G. (2013). No Control Genes required, Bayesian analysis of qRT‐PCR data. PLoS ONE, 8(8), e71448 10.1371/journal.pone.0071448 23977043 PMC3747227

[eva12773-bib-0055] McAllister, C. H. , Fortier, C. E. , St Onge, K. R. , Sacchi, B. M. , Nawrot, M. J. , Locke, T. , & Cooke, J. E. K. (2018). A novel application of RNase H2‐dependent quantitative PCR for detection and quantification of *Grosmannia clavigera*, a mountain pine beetle fungal symbiont, in environmental samples. Tree Physiology, 38, 485–501. 10.1093/treephys/tpx147 29329457 PMC5982843

[eva12773-bib-0056] Meirmans, P. (2015). Seven common mistakes in population genetics and how to avoid them. Molecular Ecology, 24, 3223–3231. 10.1111/mec.13243 25974103

[eva12773-bib-0057] Mock, K. E. , Bentz, B. J. , O'Neill, E. M. , Chong, J. P. , Orwin, J. , & Pfrender, M. E. (2007). Landscape‐scale genetic variation in a forest outbreak species, the mountain pine beetle (*Dendroctonus ponderosae*). Molecular Ecology, 16, 553–568. 10.1111/j.1365-294X.2006.03158.x 17257113

[eva12773-bib-0058] Naish, K. A. , & Hard, J. J. (2008). Bridging the gap between genotype and phenotype, linking genetic variation, selection and adaptation in fishes. Fish and Fisheries, 9, 396–422.

[eva12773-bib-0059] Narum, S. R. , & Hess, J. E. (2011). Comparison of Fst outlier tests for SNP loci under selection. Molecular Ecology, 11(S1), 184–194. 10.1111/j.1755-0998.2011.02987.x 21429174

[eva12773-bib-0060] Neale, D. B. , & Salvolainen, O. (2004). Association genetics of complex traits in conifers. Trends in Plant Science, 9, 1360–1385. 10.1016/j.tplants.2004.05.006 15231277

[eva12773-bib-0061] Nelson, C. D. , & Johnsen, K. H. (2008). Genomic and physiological approaches to advancing forest tree improvements. Tree Physiology, 28, 1135–1143. 10.1093/treephys/28.7.1135 18450578

[eva12773-bib-0062] Nunes, V. L. , Beaumont, M. A. , Butlin, R. K. , & Paulo, O. S. (2011). Multiple approaches to detect outliers in a genome scan for selection in ocellated lizards (*Lacerta lepida*) along an environmental gradient. Molecular Ecology, 20, 193–205. 10.1111/j.1365-294X.2010.04936.x 21091562

[eva12773-bib-0063] Ojeda Alayon, D. I. , Tsui, C. K. , Feau, N. , Capron, A. , Dhillon, B. , … Hamelin, R. C. (2017). Genetic and genomic evidence of niche partitioning and adaptive radiation in mountain pine beetle fungal symbionts. Molecular Ecology, 26, 2077–2091. 10.1111/mec.14074 28231417

[eva12773-bib-0064] Parchman, T. L. , Gompert, Z. , Mudge, J. , Schilkey, F. D. , Benkman, C. W. , & Buerkle, C. A. (2012). Genome‐wide association genetics of an adaptive trait in lodgepole pine. Molecular Ecology, 12, 2991–3005. 10.1111/j.1365-294X.2012.05513.x 22404645

[eva12773-bib-0065] Pavlidis, P. , Jensen, J. D. , Stephan, W. , & Stamatakis, A. (2012). A critical assessment of storytelling, gene ontology categories and the importance of validating genome scans. Molecular Biology and Evolution, 29, 3237–3248. 22617950 10.1093/molbev/mss136

[eva12773-bib-0066] Pavy, N. , Boyle, B. , Nelson, C. , Paule, C. , Giguere, I. , Caron, S. , … Mackay, J. (2008). Identification of conserved core xylem gene sets, conifer cDNA microarray development, transcript profiling and computational analyses. New Phytologist, 180, 766–786. 10.1111/j.1469-8137.2008.02615.x 18811621

[eva12773-bib-0067] Pfaffl, M. W. , Tichopad, A. , Prgomet, C. , & Neuvians, T. P. (2004). Determination of stable housekeeping genes, differentially regulated target genes and sample integrity, *BestKeeper* – Excel‐based tool using pair‐wise correlations. Biotechnology Letters, 26, 509–515. 10.1023/B:BILE.0000019559.84305.47 15127793

[eva12773-bib-0068] Plaxton, W. C. (1996). The organization and regulation of plant glycolysis. Annual Review of Plany Physiology and Plant Molecular Biology, 47, 185–214. 10.1146/annurev.arplant.47.1.185 15012287

[eva12773-bib-0069] Primmer, C. R. (2009). From conservation genetics to conservation genomics. The Year in Ecology and Conservation Biology, 62, 357–368. 10.1111/j.1749-6632.2009.04444.x 19432656

[eva12773-bib-0070] Pritchard, J. K. , Stephens, M. , & Donnelly, P. (2000). Inference of population structure using multilocus genotype data. Genetics, 155, 945–959. 10835412 10.1093/genetics/155.2.945 PMC1461096

[eva12773-bib-0071] R Development Core Team . (2017). R, A language and environment for statistical computing. Vienna, Austria: R Foundation for Statistical Computing.

[eva12773-bib-0072] Raines, C. A. (2003). The Calvin cycle revisited. Photosynthesis Research, 1, 1–10. 10.1023/A:1022421515027 16245089

[eva12773-bib-0073] Roe, A. D. , Rice, A. V. , Coltman, D. W. , Cooke, J. E. K. , & Sperling, F. A. H. (2010). Multilocus species identification and fungal DNA barcoding: Insights from blue stain fungal symbionts of the mountain pine beetle. Molecular Ecology Resources, 10, 946–959. 10.1111/j.1755-0998.2010.02844.x 21565104

[eva12773-bib-0074] Safranyik, L. , & Carroll, A. (2006). The biology and epidemiology of the mountain pine beetle in lodgepole pine forests In Safranyik L., & Wilson W. (Eds.), The mountain pine beetle, a synthesis of biology, management and impacts on lodgepole pine (pp. 3–66). Victoria, BC: Natural Resources Canada, Canadian Forest Service, Pacific Forestry Centre.

[eva12773-bib-0075] Safranyik, L. , Shrimpton, D. M. , & Whitney, H. S. (1975). An interpretation of the interaction between lodgepole pine, the mountain pine beetle, and its associated blue stain fungi in western Canada In Baumgartner D. M. (Ed.), Management of Lodgepole pine ecosystems symposium proceedings (pp. 406–428). Pullman, WA: Washington State University Cooperative Extension Service.

[eva12773-bib-0076] Salvi, S. , & Tuberosa, R. (2005). To clone or not to clone plant QTLs, present and future challenges. Trends in Plant Science, 10, 297–304. 10.1016/j.tplants.2005.04.008 15949764

[eva12773-bib-0077] Shafer, A. B. A. , Wolf, J. B. W. , Alves, P. C. , Bergström, L. , Bruford, M. W. , Brännström, I. , … Zieliński, P. (2015). Genomics and the challenging translation into conservation practice. Trends in Ecology and Evolution, 30, 78–87. 10.1016/j.tree.2014.11.009 25534246

[eva12773-bib-0078] Slate, J. , Santure, A. W. , Feulner, P. G. D. , Brown, E. A. , Ball, A. D. , Johnston, S. E. , & Gratten, J. (2010). Genome mapping in intensively studied wild vertebrate populations. Trends in Genetics, 26, 275–284. 10.1016/j.tig.2010.03.005 20444518

[eva12773-bib-0079] Small, J. , & Vierstra, R. D. (2004). The ubiquitin 26S proteasome proteolytic pathway. Annual Review of Plant Biology, 55, 555–590. 10.1146/annurev.arplant.55.031903.141801 15377232

[eva12773-bib-0080] Stapely, J. , Reger, J. , Feulner, P. G. D. , Smadja, C. , Galindo, J. , Eckblom, R. , … Slate, J. (2010). Adaptation genomics, the next generation. Trends in Ecology & Evolution, 25, 705–712. 10.1016/j.tree.2010.09.002 20952088

[eva12773-bib-0081] Steibel, J. P. , Poletto, R. , Coussens, P. M. , & Rosa, G. J. (2009). A powerful and flexible linear mixed model framework for the analysis of relative quantification RT‐PCR data. Genomics, 94, 146–152. 10.1016/j.ygeno.2009.04.008 19422910

[eva12773-bib-0082] Stinchcombe, J. , & Hoekstra, H. (2008). Combining population genomics and quantitative genetics, finding the genes underlying ecologically important traits. Heredity, 100, 158–170. 10.1038/sj.hdy.6800937 17314923

[eva12773-bib-0083] Subramanian, B. , Bansal, V. K. , & Kay, N. N. V. (2005). Proteome‐level investigation of *Brassica carinata* – derived resistance to *Leptosphaeria maculans* . Journal of Agricultural and Food Chemistry, 53, 313–324. 15656667 10.1021/jf048922z

[eva12773-bib-0084] Tamura, K. , Stecher, G. , Peerson, D. , Filipski, A. , & Kumar, S. (2013). MEGA6, molecular evolutionary genetics analysis version 6.0. Molecular Biology and Evolution, 30, 2725–2729. 10.1093/molbev/mst197 24132122 PMC3840312

[eva12773-bib-0087] Thompson, J. D. , Higgins, D. G. , & Gibson, T. J. (1994). CLUSTAL W, improving the sensitivity of progressive multiple sequence alignment through sequence weighting, position‐specific gap penalties and weight matrix choice. Nucleic Acids Research, 22, 4673–4680. 10.1093/nar/22.22.4673 7984417 PMC308517

[eva12773-bib-0088] Thornton, K. R. , & Jensen, J. D. (2007). Controlling the false‐positive rate in multilocus genome scans for selection. Genetics, 175, 737–750. 10.1534/genetics.106.064642 17110489 PMC1800626

[eva12773-bib-0089] Tigano, A. , Shultz, A. J. , Edwards, S. V. , Robertson, G. J. , & Friesen, V. L. (2017). Outlier analyses to test for local adaptation to breeding grounds in a migratory arctic seabird. Ecology and Evolution, 7, 2370–2381. 10.1002/ece3.2819 28405300 PMC5383466

[eva12773-bib-0090] Üstün, S. , Sheikh, A. , Gimenez‐Ibanez, S. , Jones, A. , Ntoukakis, V. , & Börnke, F. (2016). The proteasome acts as a hub for plant immunity and is targeted by Pseudomonas type III effectors. Plant Physiology, 172, 1941–1958. 27613851 10.1104/pp.16.00808 PMC5100764

[eva12773-bib-0091] Vasemägi, A. , & Primmer, C. R. (2005). Challenges for identifying functionally important genetic variation, the promise of combining complementary research strategies. Molecular Ecology, 14, 3623–3642. 10.1111/j.1365-294X.2005.02690.x 16202085

[eva12773-bib-0093] Vierstra, R. D. (2009). The ubiquitin–26s proteasome system at the nexus of plant biology. Nature Reviews, 10, 385–397. 10.1038/nrm2688 19424292

[eva12773-bib-0094] Vignieri, S. N. , Larson, J. G. , & Hoekstra, H. E. (2010). The selective advantage of crypsis in mice. Evolution, 64, 2153–2158. 10.1111/j.1558-5646.2010.00976.x 20163447

[eva12773-bib-0095] Voelckel, C. , Gruenheit, N. , & Lockhart, P. (2017). Evolutionary transcripomics and proteomics, insight into plant adaptation. Trends in Plant Science, 22, 462–471. 28365131 10.1016/j.tplants.2017.03.001

[eva12773-bib-0096] Wang, T. , Hamann, A. , Spittlehouse, D. L. , & Carroll, C. (2016). Locally downscaled and spatially customizable climate data for historical and future periods for North America. PLoS ONE, 11, e0156720 10.1371/journal.pone.0156720 27275583 PMC4898765

[eva12773-bib-0097] Wheeler, N. C. , & Guries, R. P. (1982). Population structure, genic diversity, and morphological variation in *Pinus contorta* Dougl. Canadian Journal of Forest Research, 12, 595–606.

[eva12773-bib-0098] Whitlock, M. C. , & Lotterhos, K. E. (2015). Reliable detection of loci responsible for local adaptation, inference of a neutral model through trimming the distribution of F_ST_ . American Naturalist, 186, S24–S36. 10.1086/682949 26656214

[eva12773-bib-0099] Whitney, H. S. (1971). Association of *Dendroctonus ponderosae* (Coleoptera: Scolytidae) with blue stain fungi and yeast during brood development in lodgepole pine. Canadian Entomologist, 103, 1495–1503.

[eva12773-bib-0100] Wikelski, M. , Spinney, L. , Schelsky, W. , Scheuerlein, A. , & Gwinner, E. (2003). Slow pace of life in tropical sedentary birds, a common garden experiment on four stonechat populations from different latitudes. Proceedings of the Royal Society London B, 270, 2383–2388. 10.1098/rspb.2003.2500 PMC1691521 14667355

[eva12773-bib-0101] Yanchuk, A. D. , Murphy, J. C. , & Wallin, K. F. (2008). Evaluation of genetic variation of attack and resistance in lodgepole pine in the early stages of a mountain pine beetle outbreak. Tree Genetics & Genomes, 4, 171–180. 10.1007/s11295-007-0098-9

[eva12773-bib-0102] Yates, C. M. , Filippis, I. , Kelley, L. A. , & Sternberg, M. J. E. (2014). SuSPect, enhanced predictions of single amino acid variant (SAV) phenotype using network features. Journal of Molecular Biology, 426, 2692–2701. 24810707 10.1016/j.jmb.2014.04.026 PMC4087249

[eva12773-bib-0103] Yemshanov, D. , McKenney, D. W. , & Pedlar, J. H. (2012). Mapping forest composition from the Canadian National Forest Inventory and land cover classification maps. Environmental Monitoring and Assessment, 184(8), 4655–4669. 10.1007/s10661-011-2293-2 21887479

