# Supplementary material for: Linking genotype to phenotype to identify genetic variation relating to host susceptibility in the mountain pine beetle system
Source: Evol Appl. 2019 Feb 19;13(1):48–61. doi: 10.1111/eva.12773 (PMC6935584; doi:10.1111/eva.12773)
Supplement: Supplementary file 1 [file EVA-13-48-s001.zip › eva12773-sup-0002-Supinfo.docx]

**Gene expression analysis of candidate genes to select stable reference genes**

**Methods**

To identify appropriate candidate genes for our expression analysis, we assessed six potential candidate genes (Table S2) under both inoculated, and wound conditions across a time course of 1 and 7 days post fungal inoculation. For additional detail on experimental setup and growth chamber conditions refer to Arango-Velez *et al.* (2015). Total RNA was extracted for qRT-PCR from 32 lodgepole and 32 jack pine individuals (∼100 mg phloem tissue per extraction). We followed the protocol of Chang *et al.* (1993) with modifications by Pavy et al. (2008), and quantified with a NanoQuant 200 (Tecan Infinite, Morrisville NC, USA). For cDNA synthesis, we used two micrograms of total RNA treated with DNaseI (Invitrogen; Life Technologies, Burlington, ON, Canada), and used Superscript II reverse transcriptase, following the manufacturer’s protocols (Invitrogen).

Primer Express (v3.0, Applied Biosystems, Mississauga, ON,Canada) was used to design primers for qRT-PCR (Table S2). Quantitative RT-PCR reactions (10 μl) consisted of master mix (0.2 mM dNTPs, 0.3 U Platinum Taq Polymerase (Invitrogen), 0.25Å~ SYBR Green, and 0.1Å~ ROX), 20 ng cDNA and 0.8 μM primers. We ran three technical replicates for each of the four biological replicates per treatment, and analyzed on an ABI PRISM 7900HT Sequence Detection System (Applied Biosciences). The cycling protocol was as follows: 95 °C for 2 min followed by 40 cycles of 95 °C for 15s, and 60 °C for 1 min. Melting curves were generated using 95 °C for 15s, 60 °C for 15 S, and 95 °C for 15s. We analyzed differences in expression across the treatments using the Ct values in BestKeeper v 1.0 (Pfaffl *et al.* 2004).

**Results**

Using the Ct values from the qRT-PCR, we assessed their stability across treatments by examining the standard deviation (SD) across all replicates. Standard deviation < 1 indicates the expression differences do not appreciably change across the treatments (Pfaffl *et al.* 2004). All genes examined had SD values less than 1 (Table S4), with three of the genes (lipid transfer protein, KNOX3, and SBPase) had SD values close to 1, while Eukaryotic translation initiation factor 5A-1 (TIF5α) and elongation factor 1 (EFIα) had the lowest stability values; these two genes were therefore selected as reference genes for our experiment.

Table S2. qRT-PCR primers to assess transcript abundance across fungal inoculation and wounding treatments to identify appropriate reference genes

| SNP locus | Clone ID | Forward Primer | Reverse Primer |
| --- | --- | --- | --- |
| Jp_C25075p377 | PBA011_E12 | GCAGTGGACGATCCCAGA | AGGACAGAGTGTTGGCAGGAA |
| Jp_c43213p458 | PBA024_N01 | CCCCCGTTTGAGTGTTACGT | GTTGGCTGGCATCACCATTT |
| JpLpc44782p470 | PBA018_N23 | CCTATGCTGAGCCTTCATTTGTT | AAAGTCAGGACAGCGTCTTGTG |
| Lodgc118p350 | PBA023_E11 | AAAGTCAGGACAGCGTCTTGTG | GCCAGTTGCTCGCCAA |
| Jp_C25075p377 | PCO0124_K14 | GGAGCCTGCAAAGAGTGTGTT | GGAATACCCTCTGCTTCGATTG |
| Jp_c43213p458 | PCO028_M18 | CCCCCGTTTGAGTGTTACGT | GTTGGCTGGCATCACCATTT |
| JpLpc44782p470 | PCO0120_M06 | CCTATGCTGAGCCTTCATTTGTT | TCCTGCTGCTGTGGAGGTAA |
| Lodgc118p350 | PCO0115_C18 | AAAGTCAGGACAGCGTCTTGTG | AGCTGCTCGTCACCAAAGGT |

Table S3. Primers designed for cloning the target genes associated with SNPs Lodgc1087 and Lodgc2304, and the primers designed from the clones for measuring transcript abundance. The “*” indicates the primers used for allelic sequencing.

qRT-PCR primers:

| SNP locus | qRT-PCR Forward Primer | qRT-PCR Reverse Primer |
| --- | --- | --- |
| Lodgc1087 | GCGGCAGCTTGAGGAATCTG | GAGTAGGAGCCTTTTTCGCAGC |
| Lodgc2304 | CTGAGTTTGCTGGATTGCGGC | GCCAACGGCTTGAGCATTCC |

Cloning primers:

| SNP locus | Forward Primer | Reverse Primer |
| --- | --- | --- |
| Lodgc1087 | TTGATCCATATACAGGTGC | ATTTATTAGTTCACTGGCTCC |
| Lodgc2304 | GTTCGATCTGAGTTTGCTGG | AATCACTTTCAGACCCTGCG |

Table S4. Estimates of standard deviation of Ct values calculated for gene expression using qRT-PCR across biological treatments of wound, and fungal inoculation in lodgepole and jack pine seedlings

| Gene | Lodgepole SD | Jack pine SD |
| --- | --- | --- |
| TIF5α | 0.30 | 0.32 |
| EFIα | 0.48 | 0.56 |
| Lipid transfer protein | 0.97 | 0.64 |
| KNOX3 | 0.82 | 0.86 |
| SBPase | 0.89 | 0.67 |

**References**

Arango-Velez A, El Kayal W, Copeland CCJ, Zaharia LI, Lusebrink I, Cooke JEK (2015) Differences in defense response of *Pinus contorta* and *Pinus banksiana* to the mountain pine beetle fungal associate *Grosmania clavigera* are affected by water deficit. Plant, Cell & Environment, doi: 10.1111/pce.12615

Pavy N, Boyle B, Nelson C, Paule C, Giguere I, Caron S, *et al.* (2008) Identification of conserved core xylem gene sets: conifer cDNA microarray development, transcript profiling and computational analyses. New Phytologist 180:766-786.

Pfaffl MW, Tichopad A, Prgomet C, Neuvians TP (2004) Determination of stable housekeeping genes, differentially regulated target genes and sample integrity: *BestKeeper* – Excel-based tool using pair-wise correlations. Biotechnology Letters 26:509-515.

Figure S1. Maximum likelihood tree of proteasome alpha subunit sequences obtained from GenBank including locus Lodgc1087 (LodgeDenovo_rep_c1087). Our locus of interest groups with high confidence to other subunit type 7 sequences rther than other subunit sequences.

Figure S2. Maximum likelihood tree of triosephosphate isomerase sequences (plastid and cystolic verions) obtained from GenBank including locus Lodg_c2304. Our locus of interest groups with high confidence to the plastid version, rather than cystolic version.
